# A new approach to enhance the appeal of the Italian territory through art: three study cases from Marche Region

**DOI:** 10.1007/s12517-020-06415-2

**Published:** 2021-01-22

**Authors:** Laura Valentini, Olivia Nesci

**Affiliations:** 1 Department of Biomolecular Sciences, University of Urbino Carlo Bo, 61029 Urbino, PU Italy; 2 Department of Pure and Applied Sciences, University of Urbino Carlo Bo, 61029 Urbino, PU Italy

**Keywords:** Geomorphosites, Poetry, Ancient music, Marche Region, Italy

## Abstract

Born from a desire to promote the landscape by integrating its origins and physical aesthetics with its naturalistic, cultural, and artistic heritage, we develop narratives about three locations in central Italy, telling them in the language of popular science, supported by the language of art. The different disciplines of science, poetry, and ancient music are applied to the same sites, producing emotional experiences where the encounter and interplay between different representations, and combinations of representations, become an expression of a place. The research introduces three geosites from the Marche Region, central Italy: *I Sassi Simone e Simoncello*, *La Grotta della Beata Vergine di Frasassi*, and the *Infernaccio* Gorge, in order to propose our multidisciplinary approach to the international public. These have been chosen for their value and charm, for their different processes of genesis and evolution, and for the cultural context and feelings they evoke. In a new approach to science communication, our study uses art in the form of music and poetry to encourage people to learn about landscapes. The paper explains the genesis and evolution of the three places, suggests trekking itineraries, includes a poem written specifically for each site, and describes a piece of ancient music and video and cultural offerings relating to each location. Our goal is to educate by fashioning a new perception of landscapes, starting with their physical beauty, and then building on scientific research in co-operation with arts, to improve what we know about their problems and weaknesses, but also about their culture and other strengths.

## Introduction

A number of studies conducted across the world over the last few decades have sought to both understand the main characteristics of our geomorphological heritage and develop methods aimed at identifying, qualifying, and managing the main landforms (O’Halloran et al. 1994; Pagès 1994; Grandgirard 1999; Aringoli and Pambianchi 2009; Coratza and Panizza 2009; Gordon 2018). The term “geomorphosites”, which is a contraction of geomorphological sites, is used to refer to areas of geomorphological heritage. Geomorphosites are therefore a type of geosite, which is a part of the geosphere that is particularly important for what is known about the history of the Earth (Panizza 2001; Reynard 2009). Reynard (2004, 2005) and Reynard et al. (2007) have suggested that the value of geosites should be distinguished at two levels: central (scientific) and additional (ecological, aesthetic, cultural, and economic), with geology adding the main scientific worth. Different groups of researchers have proposed various methodological procedures focused on geomorphosite specificities (Bruschi and Cendrero 2005; Coratza and Giusti 2005; Serrano and Gonzalez-Trueba 2005; Reynard et al. 2007; Coratza and Panizza 2009). These authors have placed the emphasis on the importance quantitative evaluation of geomorphosites in order to increase the objectivity of the selection. Parametric methods are more objective, using numerically quantified criteria and making it possible to obtain clear and replicable results (Coratza and Giusti 2005; Pereira and Pereira 2010; Mucivuna et al. 2019). Unfortunately, the qualitative method of geomorphosite selection still remains the most used method even if it implies a high degree of subjectivity, and the selection criteria are not always well explained. An approach comparing and combining the two important concepts of geodiversity and the cultural landscape has also been a matter of debate for a number of years, as both are thought to be valid tools for learning about a territory. Geodiversity is focused on physical aspects, describing the variety of geological phenomena and related processes that shape the landscape. Cultural landscape, meanwhile, is defined by the multiplicity of cultural elements that characterize a place from a modern perspective, and goes beyond most studies’ typical aesthetic-perceptive visions and monodisciplinary approaches (Sharples 1995; Dixon 1996; Gray 2004; Panizza and Piacente 2003, 2008).

Since 2002 the Italian National Institute for Environmental Protection and Research (Ispra) has created an Italian Geosites Inventory that contains computerized geosites data, to the current geographic information system, which allows storage, management, analysis, and display of the data with relation to their geographical features. In the Marche area, several researches were carried on about the main geosites (Nesci et al. 2005; Aringoli et al. 2010), but still a quantitative classification of them does not exist. The sites we are going to propose, therefore, were chosen with the qualitative method, using the same assessment criteria among them (Table 1; Pereira and Pereira 2010). The paper uses various perspectives to describe three interesting sites in the Marche Region (central Italy) that exemplify the definitions described above. Firstly, we have considered the scientific value as an essential criterion in the qualitative assessment. Based on available information on the regional setting, structural framework, climatic features, and using geomorphological mapping, we have selected the three sites among the most representative of the Marche Region. They have also other variables of the scientific criterion, above all the rarity and integrity of the geomorphological characteristics, which allow to reconstruct the geological history and the paleoenvironments. Subsequently other variables were taken into consideration which constitute the additional values (Table 1) such as cultural, ecological, and aesthetic criteria. Finally, we have taken into account the accessibility of the site and also the presence of accommodation and teaching facilities like natural reserves or regional parks. Sometimes, we have indicated their vulnerability to measure the need for protection, for the safety of visitors. For all these criteria, we propose the three case studies here reported to be classified as geomorphosites.

**Table 1:** Usual criteria in geomorphosite assessment methods (Pereira and Pereira 2010)

Scientific value criteria	Additional value criteria	Management criteria
Rarity	Cultural	Accessibility
Representativeness	Ecological	Visibility
Integrity	Aesthetics	Vulnerability
Diversity		
Scientific knowledge		

The region boasts a wide variety of landscape forms that reveal geological processes of great scientific interest and a richness of beauty and charm. This wealth is a consequence of the geological history of the Apennine chain (Alvarez 2019), which produced extraordinary contrasts of physical forms in an extremely limited space. From the very dawn of time, these places have been sites of very important human settlements, providing us with testimonies of great cultural interest. The variety of landforms (mountains, hills, and coasts) has also produced an enchanting floristic and faunal heritage. Moreover, the Marche Region still has unspoiled areas where the landscape, nature, culture, and traditions provide a great opportunity for didactic and touristic development (Aringoli et al. 2010; Valentini and Nesci 2016; Nesci and Borchia 2017; Nesci and Valentini 2019).

Unfortunately, awareness of this cultural richness and wealth is limited, because the information available to the public lacks material that packs a significant emotional punch. Rectifying this means recognizing that scientific approaches and languages are often avoided instinctively by the general public for being too technical or foreign, meaning that the messages they convey are not understood. Some forms of communication related to art, like poetry and music, directly address the emotional sphere and are able to engage us in a deep and meaningful way. Indeed, if people are open to receiving it, technical information can be communicated more effectively.

**Fig. 1:**
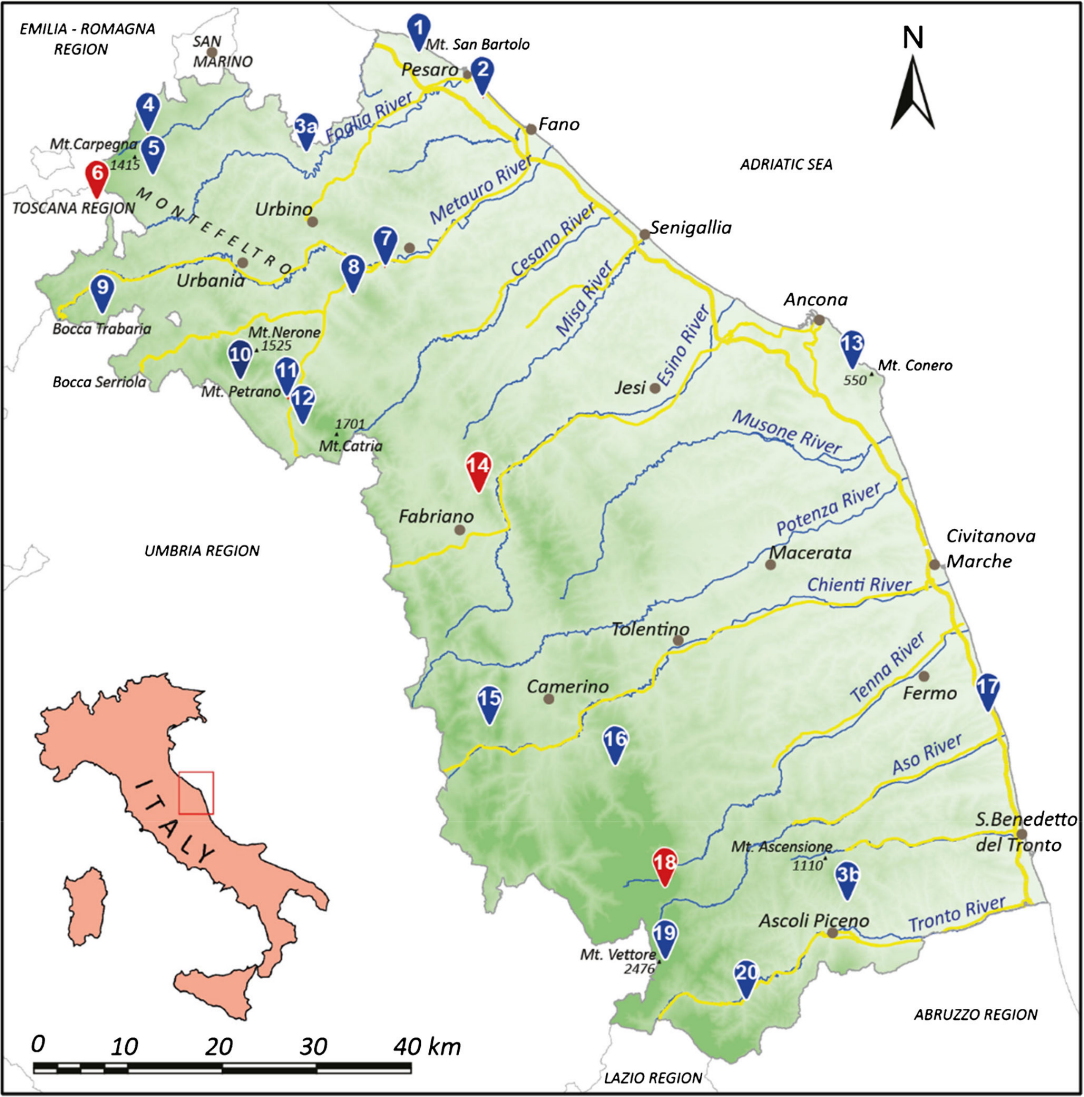
Simplified sketch of the Marche Region, depicting the 20 geomorphosites involved in the overall project. The three case studies that are the focus of this paper are shown in red (6 = *I Sassi Simone e Simoncello*; 14 = *La Grotta della Beata Vergine di Frasassi*; and 18 = the *Infernaccio* Gorge (©2020 Marche Region)

A few years ago, and linked by a common interest in the territory, we came together as a team of five researchers-artists with different skills, to which we gave the name *TerreRare* (which signifies the rare earth elements, but also refers to the areas of rare beauty in Italy), whose aim is to promote a deeper public awareness of the territory, by using three types of “language”: science, music, and poetry. The group consists of two geologists, a writer/poet, a musician/musicologist, an actor and a video-maker. We started in 2014 by proposing live events occurred in central Italy, and in parallel with these, we presented our idea in national and international scientific conferences (Nesci and Valentini 2015; Valentini and Nesci 2016; Nesci et al. 2018). The project we have created is dedicated to the development of the Marche Region in central Italy, and has the overall goal of increasing public awareness of the landscape as a resource. The whole project took place thanks to a regional announcement dedicated to the development of the Marche Region. It started in February 2018 and concluded in February 2020. The work described here is available to the public via a book in original language (Nesci and Valentini 2019) dedicated to 20 geosites in the region (Fig. 1; Table 2), and a website where the contents of the book have been summarized (https://www.terreraremarche.it). The book is also available in interactive form, in the DVD attached to the volume. The project’s poems and pieces of music provide the soundtrack to videos that use art creatively to interpret science and nature. The public can also attend live events dedicated to the landscapes that form the content of the project. More information on our communication method during the live shows is available in Nesci and Valentini 2020. At the moment, we state the importance to propose our multidisciplinary approach to the international public, since the co-operation between scientific and artistic communities originated new ways of research in the environmental matters, by stimulating the discussion and by providing emotional and human context through the arts.

**Table 2:** The 20 geomorphosites involved in the overall project, located in Fig. 1. The three case studies that are the focus of this paper are shown in bold

1	*La falesia costiera del San Bartolo*
2	*I ciottoli del Monte Ardizio*
3a	*I calanchi di Monte Calvo*
3b	*I calanchi di Monte dell’Ascensione*
4	*Le guglie di Montecopiolo*
5	*I conglomerati di Pietrafagnana*
**6**	***I Sassi Simone e Simoncello***
7	*Le Marmitte dei Giganti*
8	*La Gola del Furlo*
9	*Le Piote di Sant’Antonio*
10	*L’arco naturale di Fondarca*
11	*I Flatiron del Monte Petrano*
12	*L’anfiteatro del Tenetra*
13	*L’orizzonte del Trave*
**14**	***La grotta della Beata Vergine di Frasassi***
15	*I Piani di Montelago*
16	*Le Lame Rosse*
17	*Torre di Palme*
**18**	***La gola dell’Infernaccio***
19	*La faglia del Monte Vettore*
20	*Le sorgenti calde di Acquasanta*

The aim of this paper, therefore, is to recognize our multidisciplinary approach in the international scientific community other than highlight three geomorphosites in the Marche Region, using art to give visitors of all ages and cultural backgrounds the tools to understand a place in all its complexity (physical, cultural, and emotional landscape). Our goal is to pique the public’s interest in the genetic aspects of the territory, because it is from these characteristics that the place derives its beauty, its history, and the culture and traditions that have developed around it.

Conscious that the vulnerability of geomorphosites is related to both natural and human effects, we propose a communicative method that has the power to not only amplify the beauty of a place but also reach and engage people more directly, awakening a desire to preserve the wealth they see around them.

## Methods

We have combined three different forms of communication—science, music, and poetry—to focus on the same site and to convey the history and evolution of that landscape. Our method proceeds following two different routes. The first analyzes the landscape from a scientific point of view, attempting to explain how it has originated, evolved, and changed. Indeed, giving the public such a context enables them to recognize the potential fragility of the environment around them. The second one uses the human context to examine the landscape from a perspective more closely related to the visual and emotional impact that a place evokes: its history, its cultural significance, and its instability. The latter approach is perhaps more abstract and more intimate, and has been developed by employing forms of communication like music and poetry. When combined, these perspectives produce an emotional experience that improves the public’s understanding of the landscape in all its different respects, not only its strength but also its fragility.

Our attempts to do so always begin by describing the geomorphological evolution of a site, analyzing the processes through which it was created and then modified over time. We provide an itinerary for each location, which highlights a number of stops with particularly significant views and describes the distinctiveness of the landscape at each of them. Then, after identifying some keywords that epitomize a location’s genesis, evolution, history, or evoked atmosphere, we represent these words through music and poetry. More particularly, we try to explain the same information, but use a more direct emotional path. The musician attempts to reproduce the emotional impact of a site through a piece of ancient music for the harpsichord. The pieces have been chosen for their capacity to communicate the aspects of a place through elements that belong to the musical language. The harpsichord has an incisive and gritty tone that clearly expresses the “strength” of the landscape, while early music is well suited to representing natural forms with a history that began millions of years ago. Then, using the same ideas and words and translating them into verse, the poet dedicates specific poems to these places through metaphors that become a cognitive tool capable to link nature and thought.

Through our descriptions, the project is devoted to promoting the Marche Region’s landscapes in all their peculiarities—genetic, naturalistic, and cultural—to a wider public of all ages and cultural backgrounds. It is our view that this approach will succeed at increasing the awareness of visitors and motivating them to learn more.

There are many amazing Italian landscapes, but the focus of this paper is on three geomorphosites located in the north, centre, and south of the Marche Region: *I Sassi Simone e Simoncello*, *La Grotta della Beata Vergine di Frasassi*, and the *Infernaccio* Gorge, respectively (Fig. 1; Table 2). These sites have been chosen not only for their undoubted value and charm but also for their different origins and cultural contexts, and for the feelings that they have the power to evoke.

## I Sassi Simone e Simoncello

### The geology

**Fig. 2:**
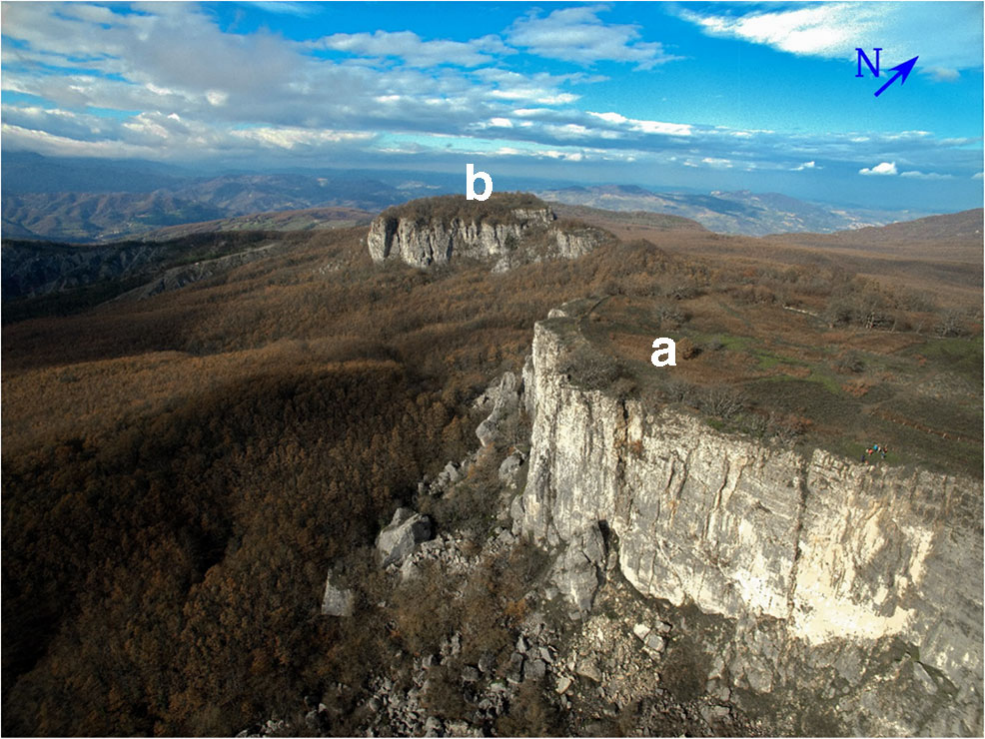
Overview of *Sassi Simone* (a) *e Simoncello* (b)

**Fig. 3:**
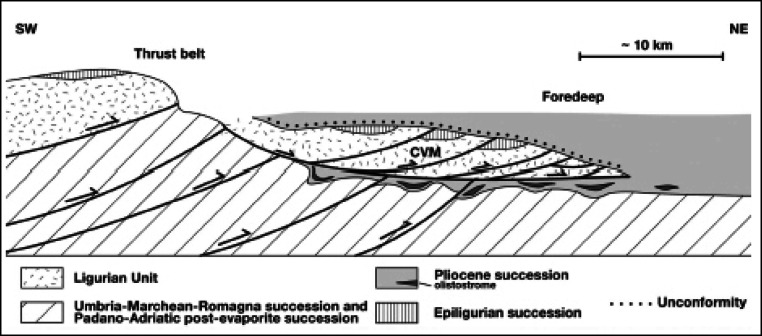
Sketch showing the relationships between the *Coltre della Val Marecchia*’s underlying successions (CVM) and contemporaneous sedimentation during emplacement (Cornamusini et al. 2017)

**Fig. 4:**
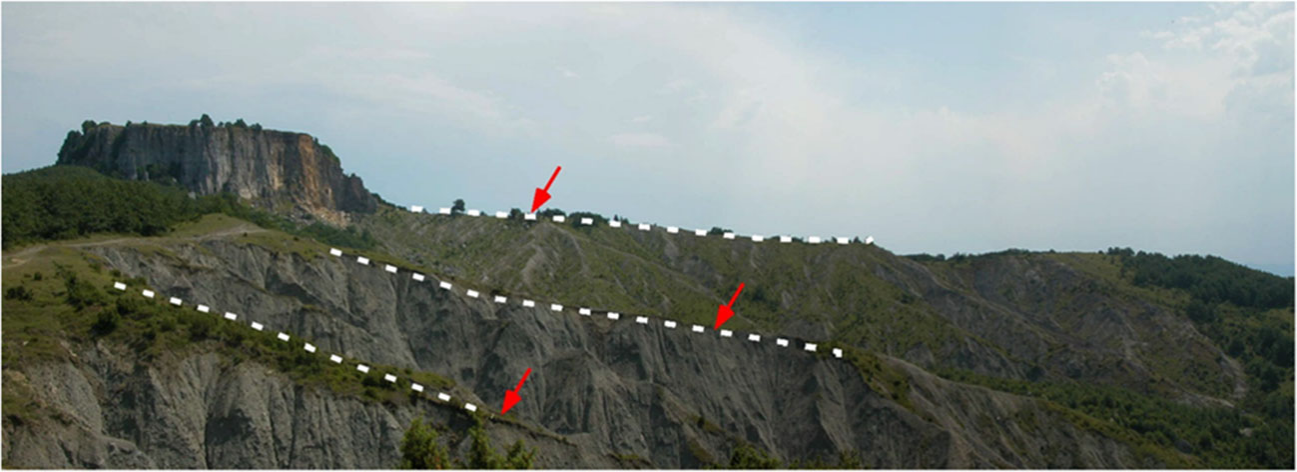
Overview of *Sasso Simone* and strips of preserved pediment (dotted line), with the boulders transported in the crests (red arrow)

**Fig. 5:**
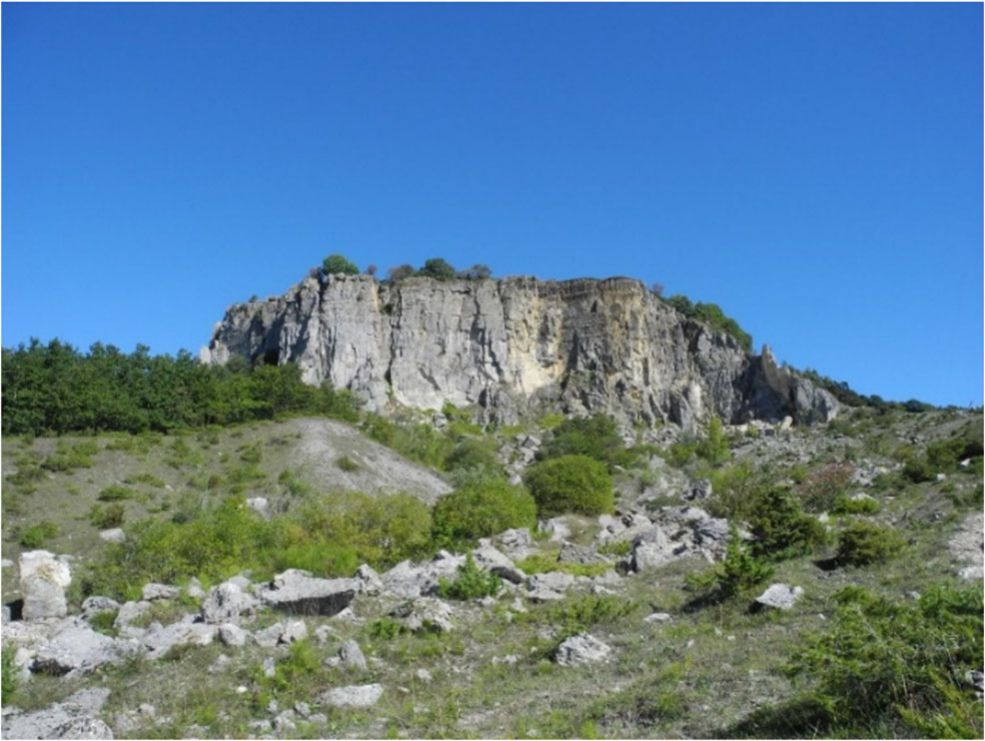
Debris flow at the base of *Sasso Simone*

**Fig. 6:**
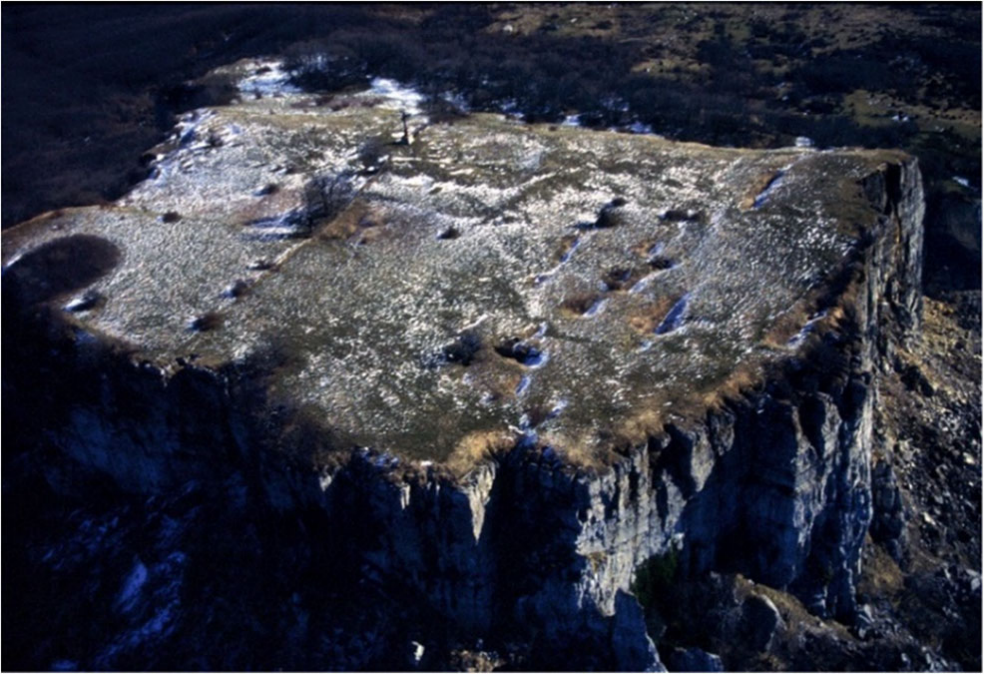
Top surface of *Sasso Simone* with deep fractures clearly visible

*I Sassi Simone e Simoncello* (Fig. 2) are two characteristic cliffs located along the Apennine ridge, between Tuscany and Marche (central Italy). Their particular tabular shape is a landmark that is not only visible from long distances but also the most significant morphological element in the Regional Park of *Sasso Simone and Simoncello*. The cliffs are part of the Val Marecchia Nappe (called *Coltre della Valmarecchia*) which is a complex stack of allochthonous and semi-allochthonous units emplaced in a foredeep basin during the Late Miocene to Early Pliocene (Fig. 3, Cornamusini et al. 2017). The Nappe is formed by Ligurian Units overlain by Epi-Ligurian ones. The Epi-Ligurian Units (Langhian-Messinian) consist of a wide variety of lithotypes ranging from conglomerates, sandstones, and biocalcarenites, to gypsum, marls, and clays. The Ligurian Units, in turn, are represented by the *Pietraforte-Alberese* succession (Maastrichtian-Eocene), where chaotic varicoloured shales are associated with more competent lithotypes such as limestones, marly mudstones, and sandstones to form a melange. In any case, whatever the mechanism of the emplacement, e.g. gravitational olisthostrome, gravitational slide, resulting of a shortening within the context of an active roof duplex (De Feyter 1991; Conti 1994; Nesci et al. 2005), from a geomorphological standpoint, the Nappe structure, because of the strong structural constraint, produced landscapes which, besides impressive natural sceneries and amazing landforms, are matters of great scientific and educational significance. *I Sassi Simone and Simoncello* are the remnants of a vast calcareous shelf deposited in a shallow marine environment in the area now corresponding to the high Tyrrhenian Sea and subsequently shifted on top of the Ligurian allochthonous Units toward the Adriatic domain during the Upper Miocene-Lower Pliocene. The two blocks are comprised of Miocene calcarenitic bodies from the *San Marino and Monte Fumaiolo Formation (Fm.)*, overlying the Cretaceous *Argille Varicolori Fm.* The intense tectonic stresses associated with the orogenetic movements of the Northern Apennines have caused the subdivision of the original calcareous shelf into several dismembered blocks, most of which have been successively dismantled by weathering and erosion. The relict pediments frequently found in this area have made it possible to identify how the landscape has been transformed, including due to climate change (Guerra and Nesci 1999). During the Middle-Upper Pleistocene cooler periods, the action of freezing and thawing on already intensely fractured rocks caused the rockfalls from the original escarpments, triggering a strong retreat of the mountain front. Soil-flow processes, sheetwash, and snow waters caused the fallen boulders to move over the periglacial pediment for long distances on the soft underlying clays. The changed climatic conditions experienced by this area in the Holocene led to the very rapid development of the drainage network, causing intense erosion that reduced the reliefs to their current size. Huge blocks of the relict pediment can still be seen over the badland crests (Fig. 4), and many debris can still be observed inside the mudflows confined in the valleys (Fig. 5). At present, the flat top of *Sasso Simone* is interrupted by deep fractures that are clearly visible on the surface (Fig. 6). Water percolation has widened the cracks, producing characteristic aligned trenches and depressed areas that constitute elements of great instability for the entire relief.

Important human settlements have influenced the history and culture of this particular landscape since the dawn of time: the first traces of the human presence in this area, also found on the summit of *Sasso Simone*, are testified by some fragments of artefacts dating back to the prehistoric era (Veggiani 1989). Later inhabited by the Umbrians until the arrival of the Romans, it was especially during the Middle Ages that the landscape, rich in cliffs and hills, was filled with castles, fortresses, and towers on the top of the rocky peaks. During the Middle Ages, the summit plain of *Sasso Simone* was the seat of a Benedictine abbey dedicated to San Michele Arcangelo, probably founded around the year 1000, during a particularly favourable climatic period. Later, the deterioration of conditions on this site located at 1204 m above sea level, prompted the monks as early as 1279 to move their residence to the nearby castle of *San Sisto*, located at a lower altitude. The abbey was abandoned in the fourteenth century, with the exception of the little church, and around 1454 Novello Malatesta began here the construction of a *castrum*, due to its significant strategic-military position. The fortress, nevertheless, was not completed due to the defeats that the Malatesta suffered at the attacks of Federico, Duke of Urbino. In 1566 Cosimo I de Medici also began the construction of a large fortress on the summit of *Sasso Simone*: a centre with a commercial and administrative function, a real fortress city. In the summit plain, there are still preserved traces of this ancient city built, the so-called *Città del Sole*, which was completely demolished in 1673 (Cherubini et al. 2007). The geological evolution and cultural heritage present here are what make this large area of *Valmarecchia* so interesting.

**Fig. 7:**
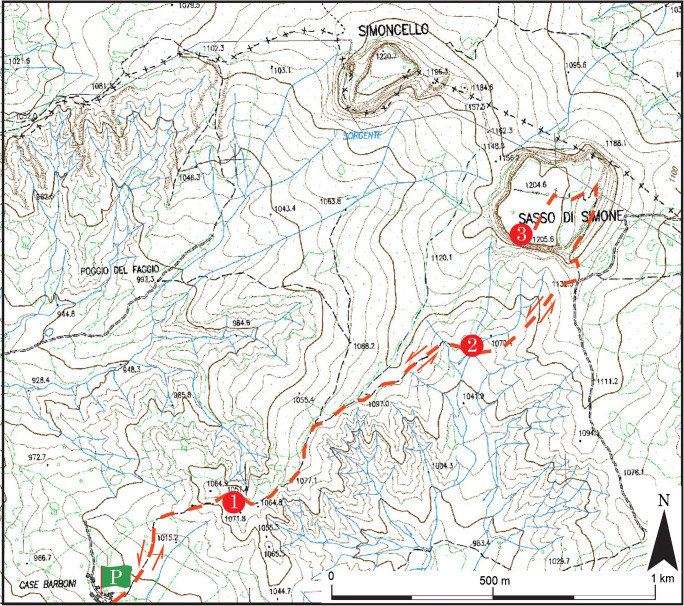
The proposed itinerary at *Sasso Simone*. P, parking area; 1, 2, and 3—stop (©2020 Marche Region)

The start of the itinerary we propose here is reachable by car from Sestino and begins in *Case Barboni*, which is a very small village located on the border between the Marche Region and Tuscany (Fig. 7). Turning right from the parking area (P) in *Case Barboni* reveals an area of uncultivated land. A walk of about 300 m across it takes visitors to a control unit and a gate leading to a well-defined pathway right to the summit of *Sasso Simone*. The view at the top is magnificent, with the various colours and multiple shapes of the clays producing a setting of rare beauty (Stop 1). *Sasso Simoncello* rises on the left, while the badlands set in the varicoloured clays can be seen on the right. *Sasso Simone* appears in all its greatness in the background, but the wide flow of debris from the rock wall (Fig. 4) expresses all its fragility (Stop 2). The itinerary ends at the summit of *Sasso Simone* (1204 m), which is reachable via the ancient medieval road. The remains of *Città del Sole* are present here, and it is also possible to see a large part of central Italy, straddling the Marche, Romagna, and Tuscany regions (Stop 3).

How can the uniqueness of this landscape be communicated through poetry and music? Our goal was to identify a number of keywords that synthesize the main genetic and process mechanisms, rather than critical issues. The words we chose to represent the morphology of the place were as follows: rock giants, red clays, and battle between the Gods.

### The poem

The poet, expressly dedicating verses to these places, makes available all that in geological processes could be difficult to understand. The poetic approach uses powerful metaphors that become a cognitive tool that connects nature and thought. This understanding fosters love for the place, not only for its scientific interest but also for the purely poetic fascination of each landscape from the depth of time.

The poet recognizes in this place the memory of the day following the battle: the apparent chaos, the clays made red by blood, the shadows of fleeing men who speak different languages. He describes the ancient battle between the Gods of Earth, five million years ago. The final effect is a mysterious, archaic, and dangerous beauty.

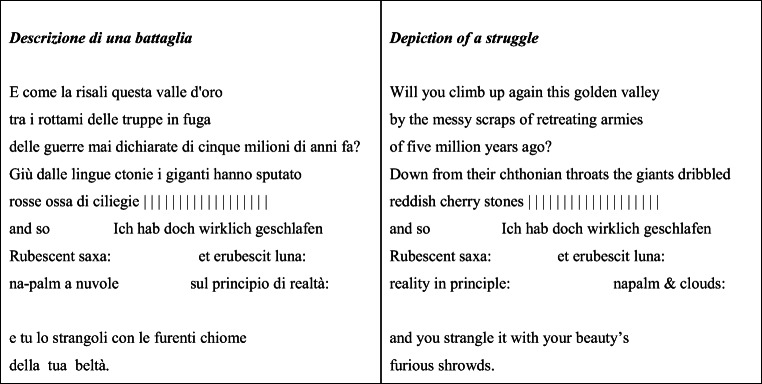



### The music

The piece of music selected to represent this site is *Jupiter* from *Pièces de clavecin*, *V*^*e*^ *Suite* in c, by Antoine Forqueray (1672–1745) (https://www.terreraremarche.it/it/db/4362/media/i-sassi-del-montefeltro).

The five suites for the viola da gamba by the French composer were transcribed for the harpsichord after his death, probably by his son, Jean-Baptiste-Antoine Forqueray (1699–1782), or perhaps his wife, Marie-Rose Dubois, who was a well-known harpsichordist.

*Jupiter* is a musical piece in the form of a rondo, organized in sections alternating with a refrain. These various segments, heavy and powerful, represent the large limestone blocks of *Valmarecchia* well, as if they are gigantic deities. Among them, *Jupiter*, the father of all the Gods, is described as peaceful, but powerful, a God who, taken over by anger (perhaps due to the bloody battles fought in these places, which are still recorded in the dark red of the clays on which the heavy blocks rest), reacts by releasing thunder and lightning, expressed in a series of energetic and virtuosistic arpeggios. He nevertheless immediately composes himself, regaining his identity with the return of the initial theme, captured only by the awareness of being the sovereign of this place of “great beauty”.

## La Grotta della Beata Vergine di Frasassi

### The geology

**Fig. 8:**
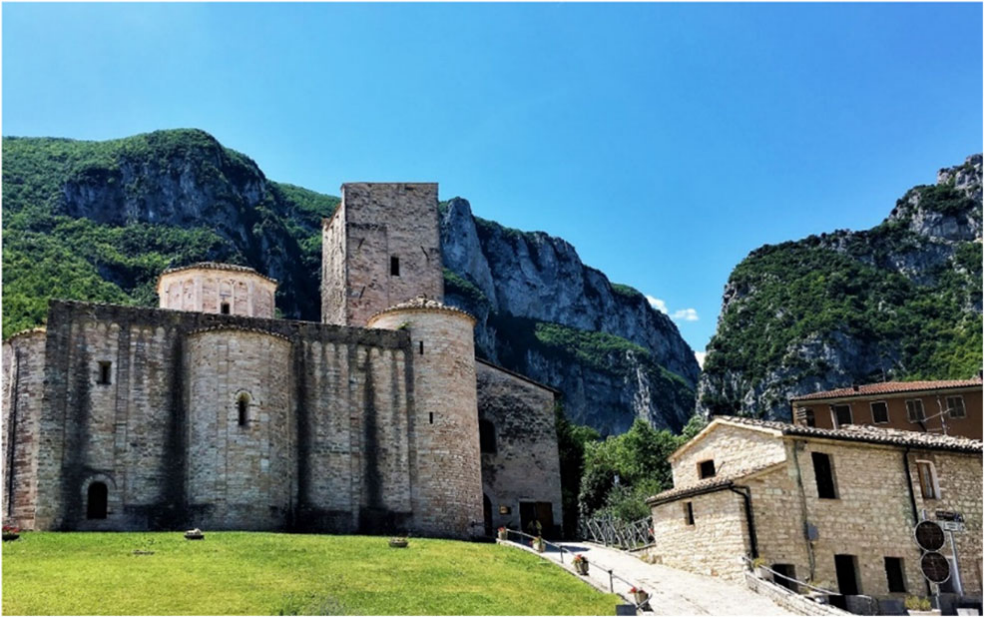
Frasassi Gorge. The Abbey of *San Vittore alle Chiuse* (eleventh century) can be seen in the foreground on the left-hand side

The Frasassi Caves, located in the Marche Region (central Italy) on the Adriatic side of the Apennine Mountains, 40 km from the coast (Fig. 1), are one of the most famous Italian underground systems, due to their importance as a show cave. There are karst systems on both sides of Frasassi Gorge, which is a 500-m-deep and 2-km-long canyon cut through by the River Sentino from west to east (Fig. 8). Elevations range between 200 m at river level and 957 m at the top of the gorge. The caves originate from the interaction between the limestone and the acidic waters ascending from a deep aquifer. Witnesses to this ascent are the sulphurous springs visible on the right bank of the River Sentino, near to the tourist entrance of the *Grotta Grande del Vento*, which are used in the *San Vittore di Genga* thermal spa. The discontinuities of the rock, represented by fractures and stratifications, are the preferential routes for the movement and action of these waters, favouring the formation of caves. The steep cliffs of the gorge clearly show the geological structure. An asymmetric anticline fold, with a main NNE vergence, was formed in the Late Miocene during a tectonic compressive phase that also caused the Apennine uplift and emersion. The eastern limb is cut by a system of N–S faults. The caves have mainly developed in the *Calcare Massiccio Fm.* (Early Jurassic), which constitutes the core of the anticline and outcrops across the entire gorge and in the hinge zone.

**Fig. 9:**
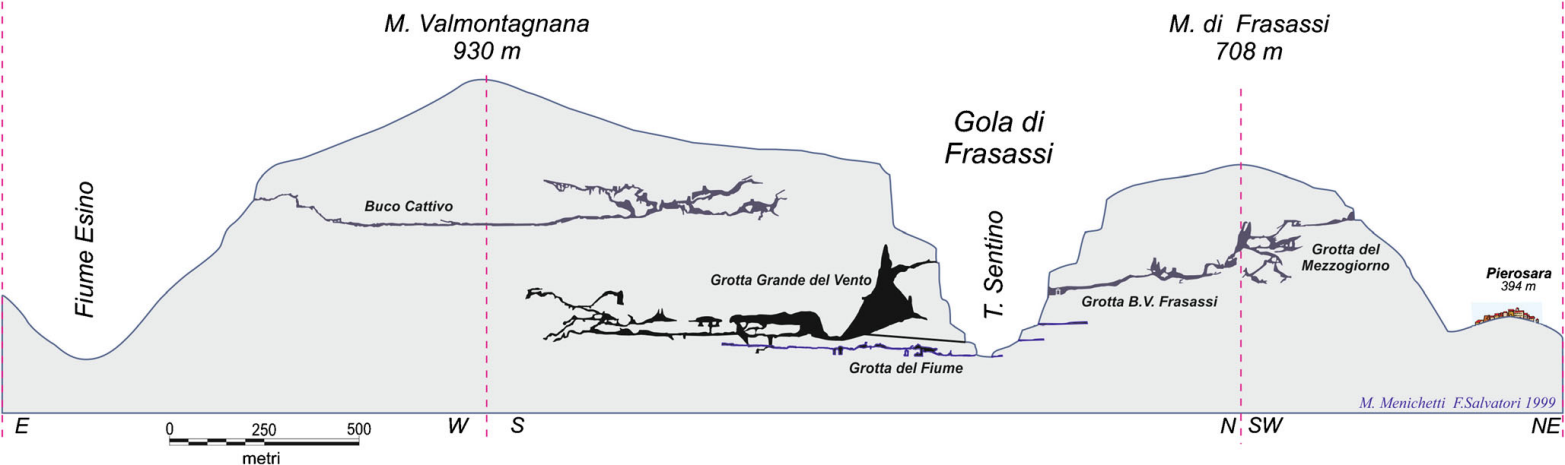
Simplified scheme of the underground systems of the Frasassi Gorge (after Galdenzi and Menichetti 2002)

**Fig. 10:**
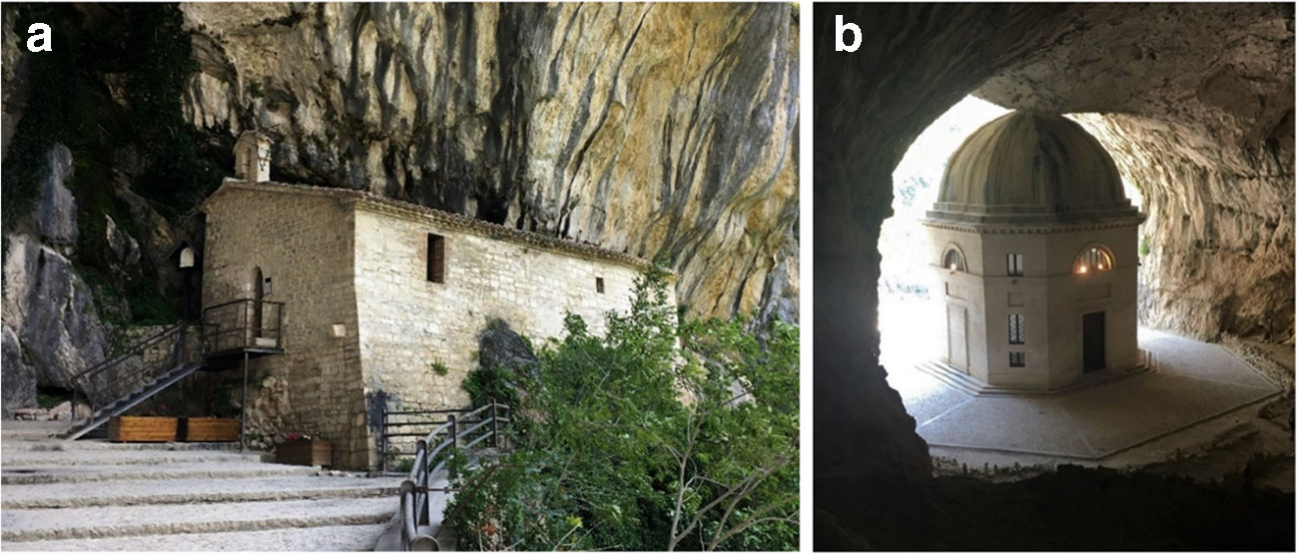
*Grotta della Beata Vergine di Frasassi*. (a) Hermitage of *Santa Maria infra Saxa*; (b) Sanctuary-church of the Valadier

**Fig. 11:**
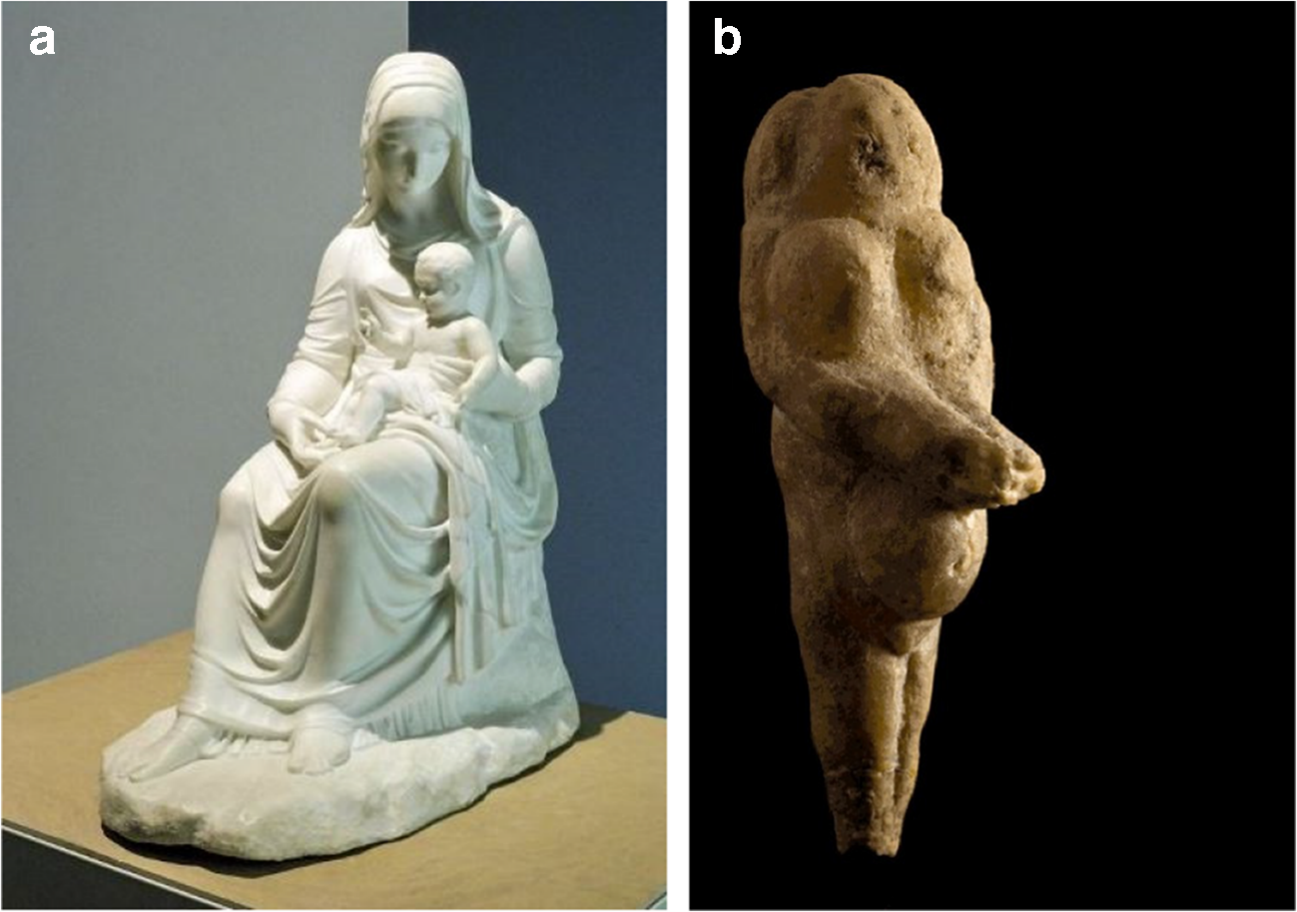
The female figure is recurring: (a) Original of the Canova sculpture (nineteenth century), Genga Museum (Marche Region), visible in a copy inside the church; (b) Venus of Frasassi found inside the cave in 2007 and now preserved in the Archaeological Museum of the Marche Region

*La Grotta della Beata Vergine di Frasassi* is a very interesting natural cavity from an archaeological point of view, given the effects of a prolonged period when it was frequented by man in protohistoric and historical times. The cave is located on the left side of the gorge (coming from Genga), at an altitude of 319 m a.s.l. and 110 m above the River Sentino, and has a magnificent entrance in the limestone wall. The cave is part of the karst complex of the *Grotta del Mezzogiorno*, the access to which can be found at about 490 m a.s.l. on the rocky cliff of the south-eastern slope of *Monte di Frasassi* (Fig. 9, after Galdenzi and Menichetti 2002). The name of the cave comes from the presence of two Christian places of worship: the hermitage of the cloistered Benedictine nuns of *Santa Maria infra Saxa* (Fig. 10a), set against the southern outer wall of the cave, and the nineteenth century church (Fig. 10b), located in the entrance hall, whose construction required levelling and widening of the entrance. The church was built entirely of travertine in 1828 by Pope Leo XII, probably designed by the architect Giuseppe Valadier. Inside, on the alabaster altar, there is a copy of the statue of the Virgin and Child in white Carrara marble, which is attributable to Canova (Fig. 11a). The excavation of the 8- to 10-m-thick sediment from the cave’s entrance hall led to the removal of the Middle Pleistocene fluvial terrace and the more recent overlying debris with anthropic traces. There is an 8-cm-high statuette, called the Venus of Frasassi (Fig. 11b), above this debris in the entrance hall. This was found inside the cave in 2007 and, with its style and proportions, falls within the Venus of Gravettiano typology (28–20,000 years ago); the morphostratigraphic data have allowed us to establish a pre-Holocene age for the statuette. The area was frequented by man for a long and discontinuous period, which persisted throughout the Bronze Age, and even in the early Iron Age (about 3000 years ago). The relics found, including a dagger and a glass-paste button, suggest the cave probably had votive and cult functions (Pignocchi and Montanari 2016). More recently, a necropolis found in the entrance hall, which was later emptied for the construction of the church, reveals that the cave also had a funeral function in the late ancient and early medieval ages.

**Fig. 12:**
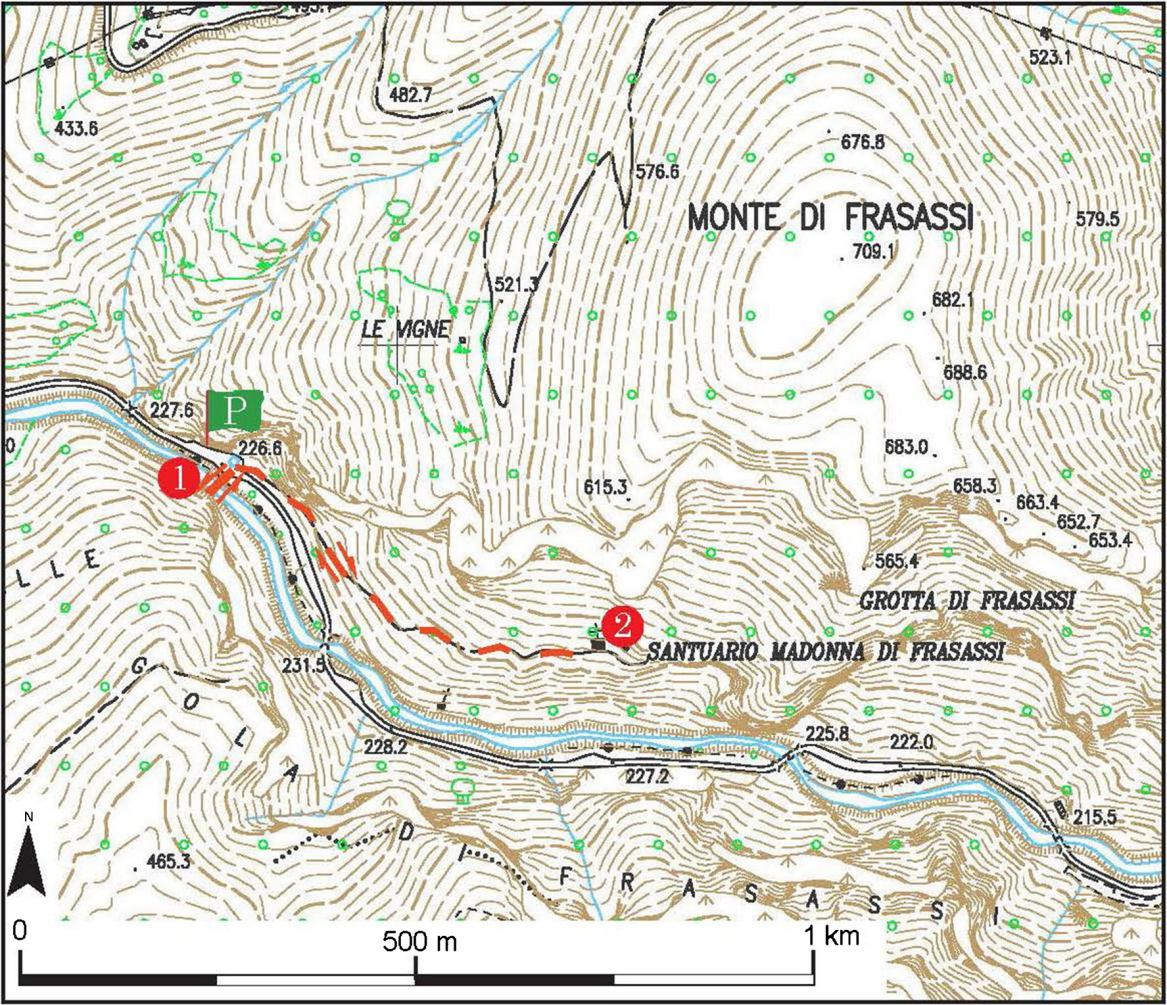
The proposed itinerary at *Grotta della Beata Vergine di Frasassi*. P, parking area; 1 and 2—stop (©2020 Marche Region)

The second itinerary relates to the *Grotta della Beata Vergine di Frasassi* and starts at *San Vittore delle Chiuse*. From there, a road across the gorge for about 2 km takes visitors to a parking area on the right (P, Fig. 12) and a path leading down to the river (Stop 1). The peace there, before the climb to the cave, is so great that it will introduce you to the place of rare suggestion that you are going to visit. Coming back to the parking, a steep road up toward the cave can be easily found. This route, which is about 700 m in length and is not difficult terrain, provides superb views of the Frasassi Gorge. The opening to the large cave appears suddenly after a walk of about 20 min, with the Valadier sanctuary in the centre (Stop 2).

At this point, the question asked earlier about *I Sassi Simone e Simoncello* is posed again: how can the uniqueness of the place be communicated through art? Here, the keywords chosen to portray the gorge through poetry and music are as follows: karstism, mother, weeping of the Earth.

### The poem

The following verses are the interpretation offered by the poet for this place: each cave is a Cave of the Nativity, where rock and water encircle and reveal life. Pining and pain are a prelude and necessary condition for life, and nothing and nowhere touches this timeless law.

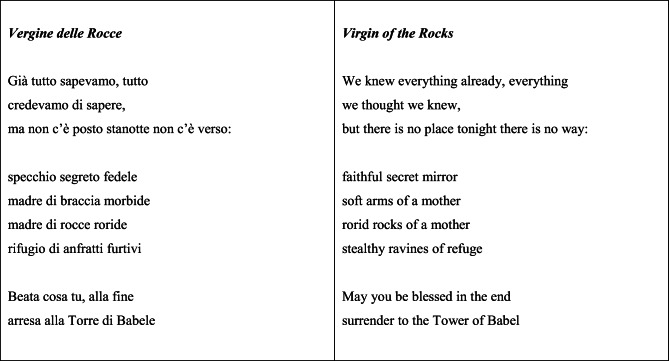



### The music

The musical piece chosen to depict this very suggestive place is *Pavana Lachrimae*, SwWV 328 (https://www.terreraremarche.it/it/db/4367/media/la-grotta-della-beata-vergine-di-frasassi), by the illustrious Dutch composer Jan Pieterszoon Sweelinck (1562–1621), and it is a variant for harpsichord of “Flow, my tears, fall from your springs”, a piece for lute and voice by the English composer John Dowland. Originally written to be purely instrumental, and given the name *Lachrimae pavane* in 1596, it is Dowland’s most famous composition. Indeed, he was so bound to it that he sometimes even signed his name as “Jo Dolandi de Lachrimae”. The piece is probably the most popular melody from the early 1600s, and was one of the favourite improvised motifs throughout the seventeenth century. There are numerous versions with different arrangements, which can be found in over 100 manuscripts and prints.

The piece was chosen for several reasons. First, the melody, intense and forlorn, is particularly suitable for expressing the aura of this sacred place. Yet, more than this, the words, which begin with tears that flow from eyes defined as “a source”, articulate the pain of the Virgin toward her son, but also of Mother Earth toward humanity. The composition immediately seems to understand and absorb the extraordinary caves created by the karst phenomenon, building up to reflect a slow, but inexorable, cry that not only consumes rock and generates cavities but also is capable of regenerating in the caves’ magnificent stalactites and stalagmites.

## The Infernaccio Gorge

### The geology

**Fig. 13:**
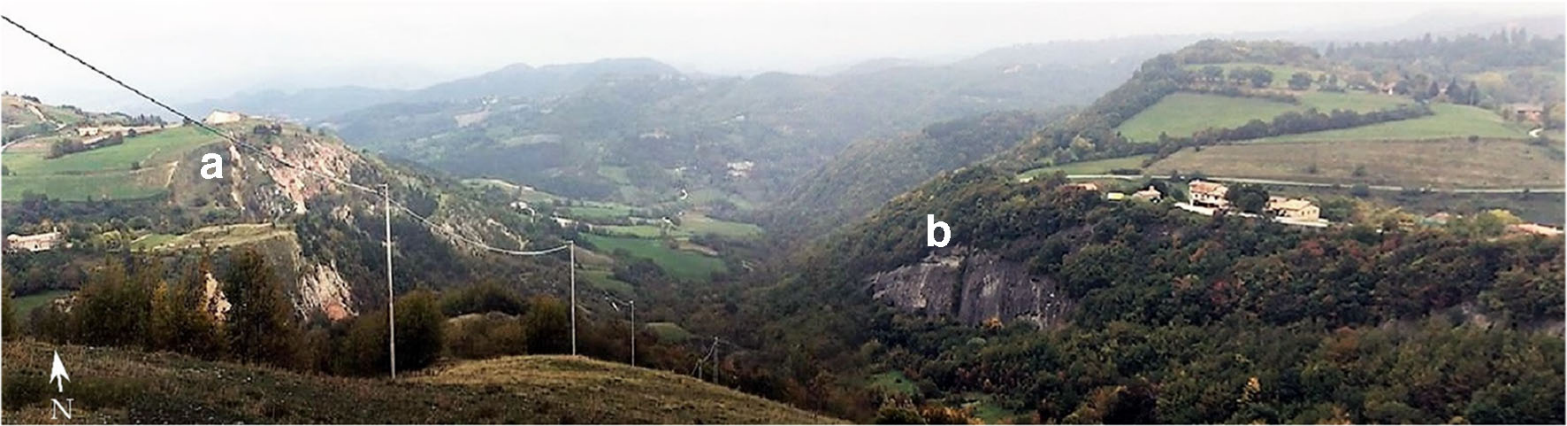
An overview of the thrust front of Mount Sibilla. The most ancient terrains (Paleogene, a) overlaid on more recent ones (Miocene, b)

**Fig. 14:**
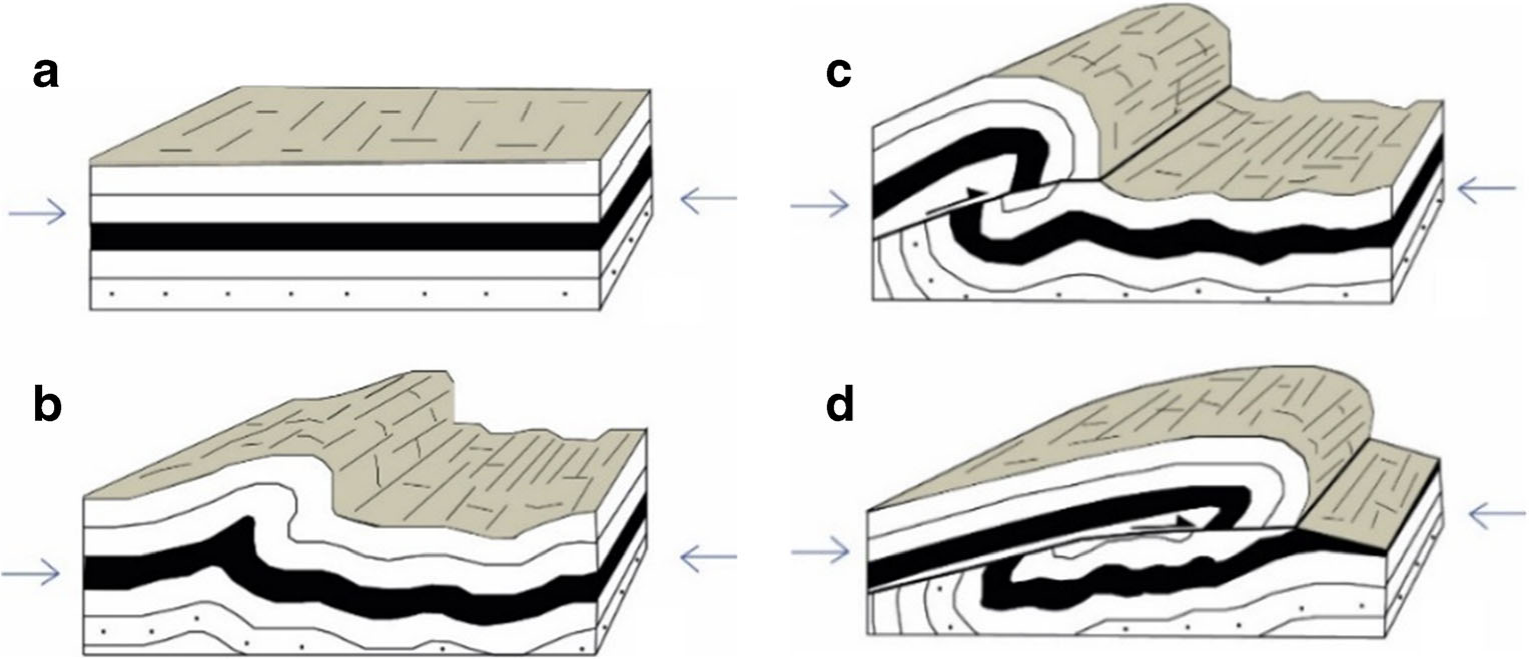
Simplified scheme of the thrust. a, b, c, and d indicate the evolution phases of the over-sliding front following tectonic compression

**Fig. 15:**
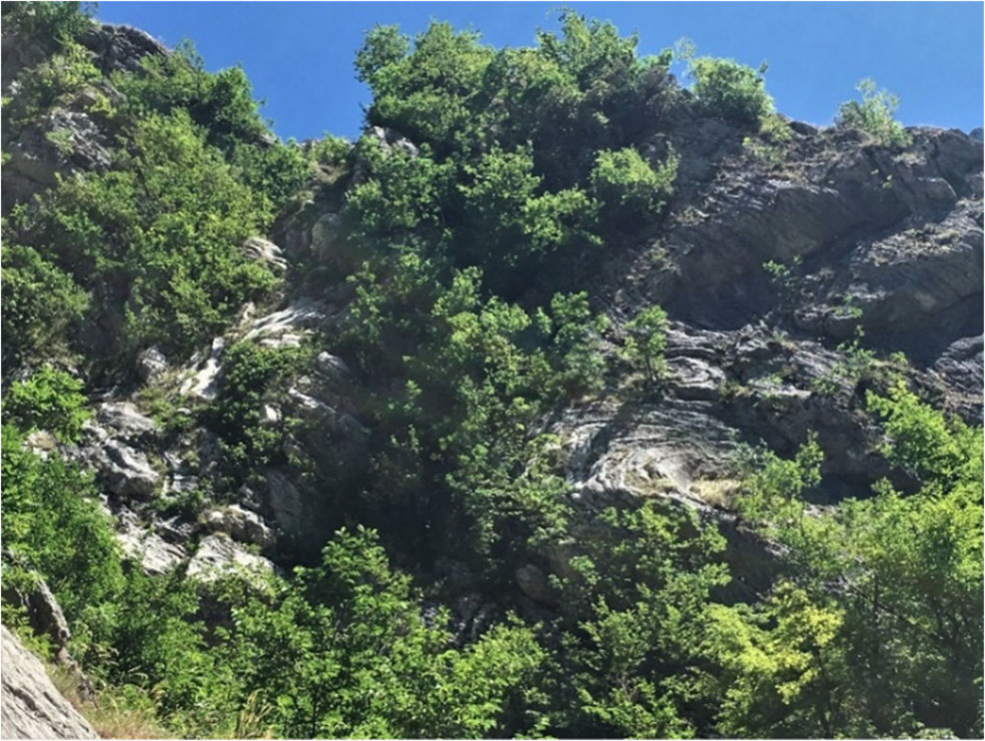
*Infernaccio* Gorge. Twisted and deformed layers on the thrust front

**Fig. 16:**
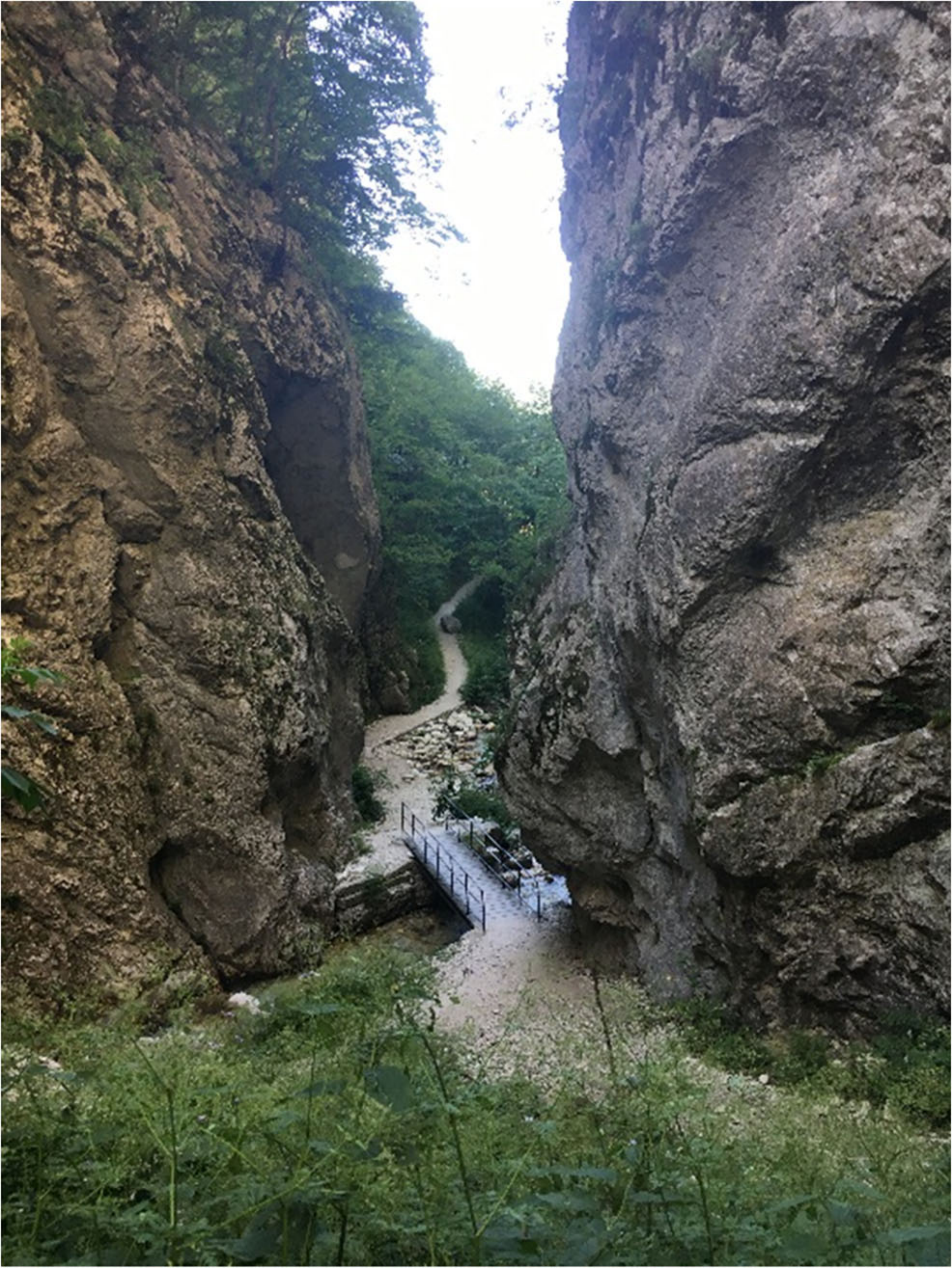
The entrance to the *Infernaccio* Gorge

The Sibillini Mountains are the steep and wild reliefs of the southern Umbria-Marche Apennines (Fig. 1). The highest peaks and most spectacular landscapes are on the Marche side and are particularly enjoyed by experienced hikers for their great scenic and naturalistic appeal. The *Infernaccio* Gorge consists of a deep incision carved by the Tenna River, and is elongated and embedded between the Sibilla (2174 m) and Priora Mounts (2332 m). The morphology of the Umbrian side is gentler and is typified by vast highlands like the plain of *Castelluccio di Norcia*. The tectonic structure characterizing this area is the Sibillini Mountains’ thrust, which limits the eastern part of the Apennine ridge (after Pierantoni et al. 2013). The Sibillini mountain chain is a NE verging foreland fold-and-thrust belt, the southernmost part of the Northern Apennine that developed during the late Miocene. The Sibillini Mountains area makes it possible to see the effects of the compressive tectonics of the Upper Miocene-Pliocene that led to the formation of the Apennines (Fig. 13). Here, strong tangential tectonic forces have caused vast parts of the Earth’s crust to advance for many kilometres above other portions, creating a stacking of older layers on younger layers (Fig. 14). The innermost region (the *Castelluccio di Norcia* basin), on the contrary, has been experiencing extension since the late Pliocene, expressed by a set of extensional faults that crosscut compressional structures forming several intramountain basins (Aringoli et al. 2016). The thrust structures show an arcuate shape traced by different stepped segments rotated from NW-SE in the northern sector to N-S in the centre and to NE-SW in the southernmost part. The hanging-wall fold structures are characterized by an axial trend parallel to the orientation of the thrust planes, while the footwall structures maintain a NW-SE axial direction. The kinematics of the thrust indicates a complex dextral transpressive component toward ENE that probably reactivates pre-existing Mesozoic structures. The orogenetic thrust, with Adriatic vergence, was so intense it produced a series of folds that overlaid on the younger formations (*Scaglie Fms.* and *Laga Fm*., in the northern and southern sectors, respectively). The outermost thrust front is well exposed near the *Infernaccio* Gorge. The river carves a large anticline just before the entrance to the gorge, with the *Scaglia Rossa Fm.* at its core, followed by the *Scaglia Cinerea Fm.* The presence of the thrust front is clearly evidenced by the strongly tectonized outcrops of the *Scaglia Rossa Fm.*, and by overturned and verticalized layers of the *Maiolica Fm.* (Fig. 15). A portion of the *Calcare Massiccio Fm.* overlies this latter formation, where the narrowest part of the gorge is engraved with smooth and vertical walls (Fig. 16). The upper part of the gorge is wider, and the landscape changes into gentle slopes used for pasture, while the valley floor remains covered by debris deposits from the sector upstream.

**Fig. 17:**
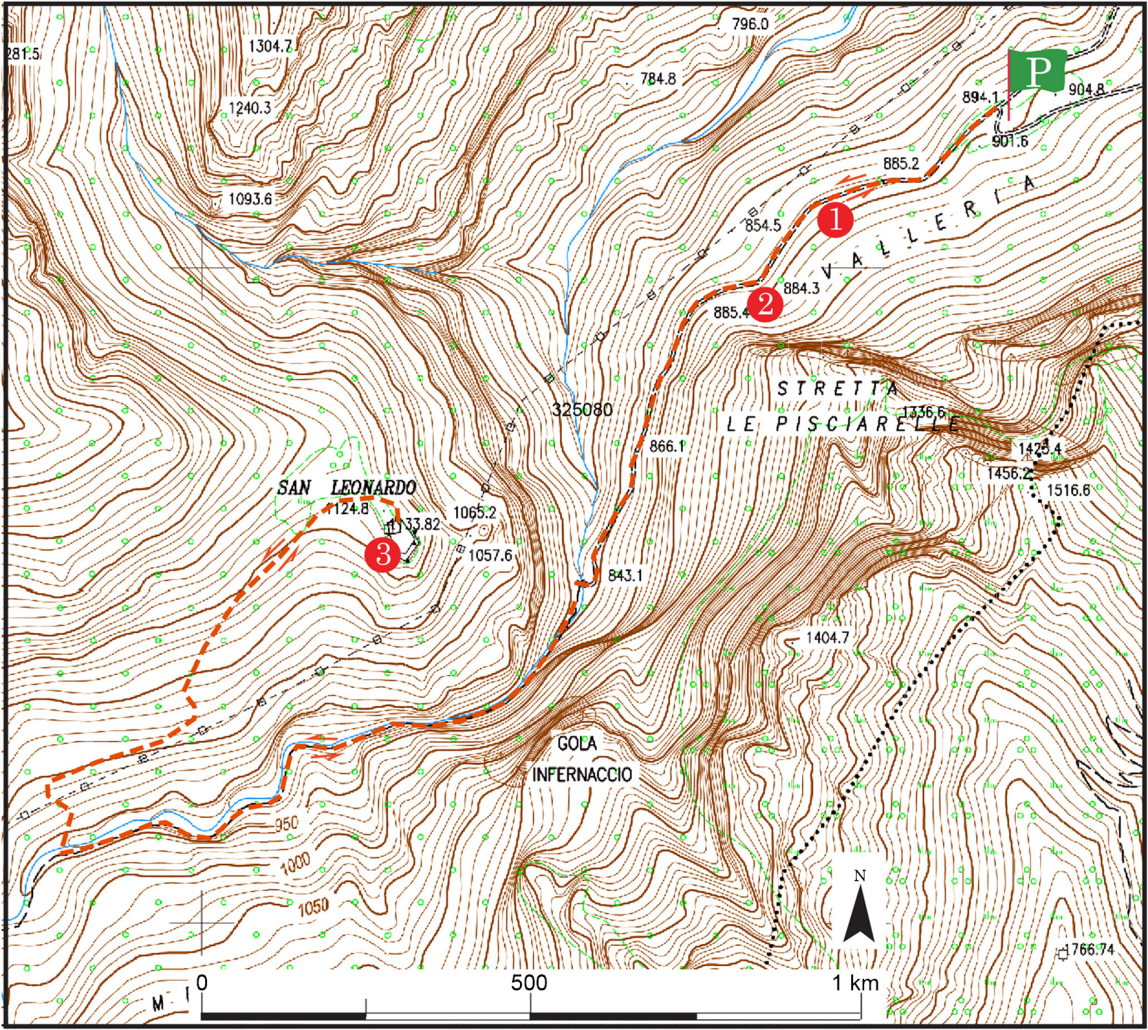
The proposed itinerary for the *Infernaccio* Gorge. P, parking area; 1, 2, and 3—stop (©2020 Marche Region)

**Fig. 18:**
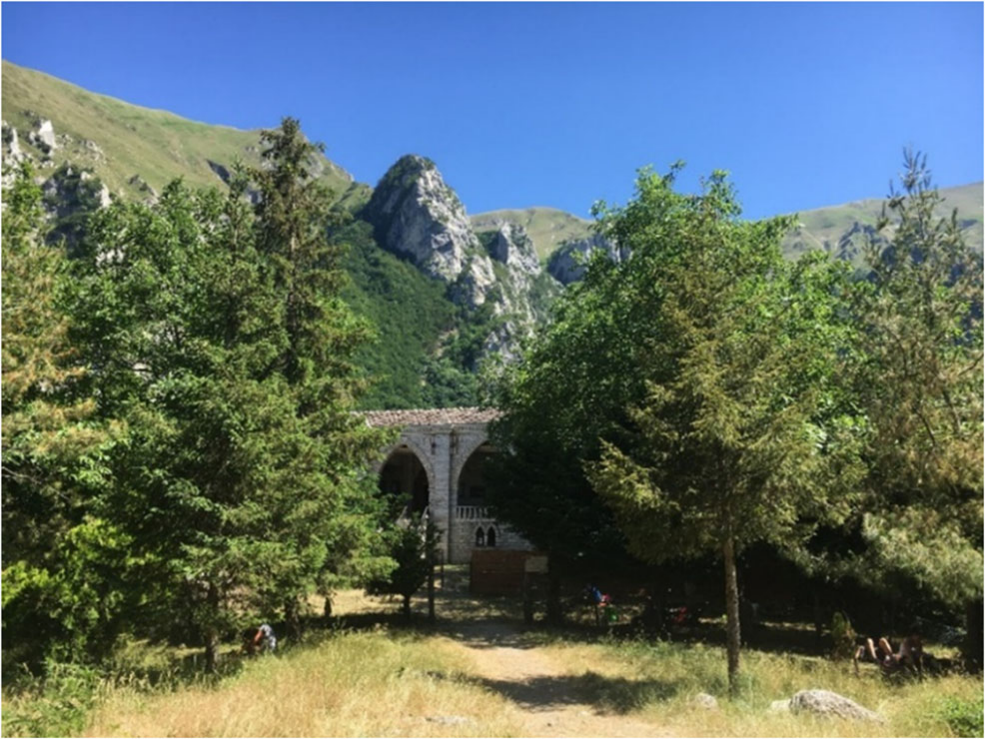
Hermitage of *San Leonardo* at the top of the proposed itinerary for the *Infernaccio Gorge*

The itinerary for the third geomorphosite begins at a small rest area (loc. Valleria), with parking (P, Fig. 17), along the dirt road from the town of Rubbiano. A rocky wall with strongly tectonized layers can be seen on the left after a few 100 m (Fig. 15). This is in correspondence with the thrust front, where the oldest Umbro-Marchigian rock sequence dates back to the most recent (Stop 1). Moving forward, one of the most spectacular stops on the route is the Pisciarelle Cave, which is a steep wall traversed by small waterfalls that must be negotiated to reach the entrance to the gorge (Stop 2). Crossing a bridge here leads to the start of an uphill path, which is the narrowest and wildest stretch of the route. The winter period should be avoided, due to the risk of landslides. After a walk of about 50 min, a path on the right leads to the extraordinary and unspoiled Hermitage of *San Leonardo* (1128 m), which overlooks the gorge and provides a magnificent view of the surrounding mountains (Fig. 18; Stop 3). San Leonardo is an ancient monastery, the oldest spiritual settlement in the Marche Region. Some archaeological research dated its origin to the second-third century BC. The main reason of the importance over the centuries is due to its location along one of the shortest passages that connected the Adriatic Sea to Rome. In this remote and hardly accessible place, as a consequence, a life that has greatly influenced our history has been developed. The Camaldolese monks lived here from the twelfth century, and transformed the place into a centre of faith, culture, and economic development, frequented by pilgrims.

The entire route (round trip) takes about two and a half hours. It is also possible to extend the route from the hermitage to the *Fosso del Rio* waterfall. Alternatively, continuing from the gorge toward the *Capo Tenna* basin leads to the upper part of the Sibilla Mount. Finally, the keywords chosen to communicate the main features of this site through poetry and music are as follows: compression, thrust, and ascent.

### The poem

Walking upward means taking an initiatory path: the ascent after the catabasis does not contemplate certainty, but demands faith, and obliges us to look up. The reward is to return to the surface to breathe, like a diver.

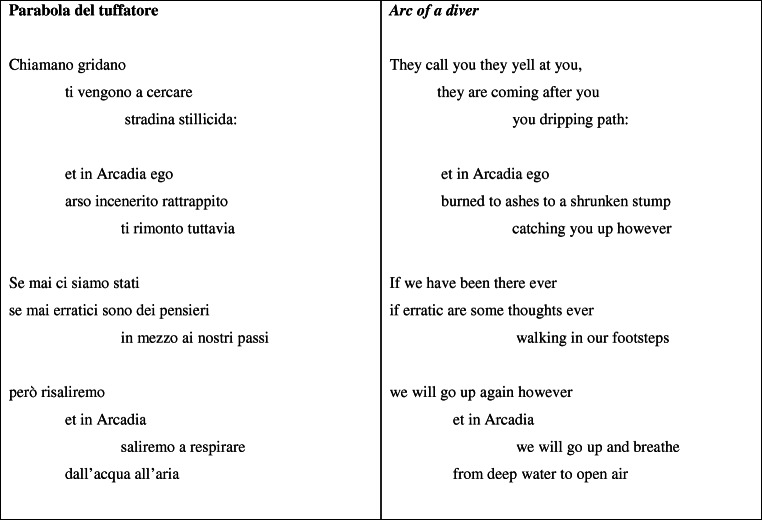



### The music

The piece of music selected to represent this very suggestive place is the *Ciacona* in e BuxWV 160, by Dietrich Buxtehude (1637–1707) (https://www.terreraremarche.it/it/db/4378/media/lorrido-dellinfernaccio) in a transcription for two harpsichords by Don Simons. Dietrich Buxtehude, a man of culture, a poet, a composer, and a virtuoso musician, was also the master of the great Johann Sebastian Bach. His organ compositions are considered to be the best of the seventeenth century’s German Organ School. This *Ciacona* in e minor is an obstinate bass whose perpetual motion is accompanied by a rich melodic elaboration: a sound building that becomes progressively more solid, up to the vigorous finale. Originally written for the organ, it is proposed here in a version for two harpsichords, where the two parts, almost equivalent, move one on top of the other: the first harpsichord has a short melodic section, while the second only reinforces the melody with the bass; then, in the next section, the roles are reversed. This mechanism is replicated throughout the piece, and the final effect is that of very similar sections that overlap alternately. This is a musical process that re-proposes what has happened to the rocky layers in the thrust of the Sibillini Mountains. This execution is carried out by a soloist who, by means of an overdubbing technique, overlaps herself.

## Discussion and conclusions

Since the user of the geomorphosites has not to be only a man of science, it is extremely important to identify and use a language that is suitable for reaching a generic public. Moreover, being the vulnerability of geomorphosites related to both natural and human effects, we adopted a communicative method that has the power to reach and engage people more directly, awakening a desire to preserve the wealth they see around them. The different elements of the nature of a place, finally, can be illustrated more effectively when a multidisciplinary method is used. This is because the project’ outputs are always multidisciplinary, and start from the genetic and evolutive history of a place, before moving through its naturalistic aspects to finally experience its cultural elements.

In the past, the communication of environmental subjects has been addressed mostly through traditional educational methods. But the communication of information through the emotional sphere is recognized to be much more effective than traditional methods. There are increasing evidences, in fact, that science-art collaborations can play a great role in this context by creating new ways of research appropriate in stimulating the emotional and human sphere. Art has a great power and provides a remarkable means to communicate specific subjects, an opportunity not to be overlooked: by addressing the emotional sphere, indeed, art manages to engage the observer in a profound and passionate way. Accordingly, we have adopted a science-communication approach that uses poetry and ancient music to encourage people to learn about these landscapes: a multidisciplinary perspective which promotes the communication of rigorous and complex scientific content related to the geological genesis and evolution of a place.

The three geomorphosites that are the focus of this paper represent different and important geological aspects of the Marche Region. The area’s physical conformation has given it a huge variety of landforms, as well great wealth in its naturalistic, historical, and cultural features; indeed, so much so that it could be viewed as an open-air museum. The geological evolution of the areas of *Sassi Simone and Simoncello*, *Grotta della Beata Vergine di Frasassi*, and *Infernaccio* Gorge, together with their cultural heritage, are so interesting that they could be considered three important geomorphosites. Our territory is a great wealth, which must be understood if it is to be loved and protected. For this reason, we decided to promote our message through different ways, involving more directly the public. Rather than just promoting the book and website, we have created live shows about the most interesting geomorphosites in the region, in which the scientific, poetic, and musical languages are combined. An initial geological-geomorphological description of the site forms the start of a trekking itinerary that also recommends various stops along the way with the most spectacular views. We deeply believed in the importance to identify a key to interpretation of the place, namely some keywords to remark to the public, which can be expressed through music and poetry. Following this interpretation, the public is involved through the reading of a poem that has been written specifically for a particular landscape, after which a piece of music from the late Renaissance or Baroque periods, performed at live on the harpsichord, is proposed. Together, these art forms are able to touch directly the public, accentuating the uniqueness and very essence of a place from both the geological-evolutive and aesthetic-cultural points of view.

The above described working method (Nesci and Valentini 2019, 2020) was applied to twenty places in the Marche Region, and the three sites here given as an example offer the reader the opportunity to appreciate a teaching method that is proving great results. The venture described in this paper has already been presented, within and out of the Marche Region, in the form of live shows, with the audience’s emotional participation in them evidencing the effectiveness of art in transmitting scientific and cultural themes, such as the origin of the landscape, the culture and traditions that the place has generated, the problems in the protection and the conservation of the place. The live shows are organized following the multidisciplinary communication method described above, combining science, projections of images and videos, recitation of poems, and live musical performances. This project is still very young; the book and the web site are concluded in full covid-19 period, when would been the best moment to promote our work with a number of live shows. Now we are starting again to have contacts with the public, and the next step of our work will be the collection of data about the response of the public. We still did not collect numerical data on the characteristics (i.e. age and cultural background) of the public that showed interest on our work. We did not still organize a systematic way to collect the appreciation of the participants. Our experience, at the moment, is based on decades of teaching at university and out of the university on topics related the environment and the territory. In the past we always used the traditional scientific communicative method and we can affirm that, introducing the arts, the involvement of people changes deeply: the public shows systematically a strong interest, rarely shown by using the traditional methods. At the end of our live shows, we are always surrounded and contacted by people, asking details of the project, communicating us their desire to better know the places and preserve them. Our videos, uploaded to the website, indicate in total about 27,000 views, and through the socials, people showed us their appreciation. The project does not provide a simple guide to the visiting of some places, but it represent a new reading of the landscape devoted to curious people, which love the territory in its morphology and its genetic aspects, which are interested to the arts and the culture which developed around the places. The project appeals to people of all ages and backgrounds, and aims to arouse in them an awareness of the cultural heritage that the territory represents, increase their understanding of the fragility of landscapes, and fuel a desire to protect and conserve this heritage. In conclusion, our method could represent a new way of teach people to perceive a different landscape, starting with its beauty but going on with the knowledge of its origins, uniqueness, history, culture, and fragility. In addition, it can represent an approach to be followed, to be improved or to be compared with different methods, addressed scientists-artists which believe in such communicative approaches.
